# Dispersal and reproductive careers of male mountain gorillas in Bwindi Impenetrable National Park, Uganda

**DOI:** 10.1007/s10329-019-00718-z

**Published:** 2019-03-07

**Authors:** Martha M. Robbins, Moses Akantorana, Joseph Arinaitwe, Peter Kabano, Charles Kayijamahe, Maryke Gray, Katerina Guschanski, Jack Richardson, Justin Roy, Vastine Tindimwebwa, Linda Vigilant, Andrew M. Robbins

**Affiliations:** 1 Max Planck Institute for Evolutionary Anthropology, Deutscher Platz 6, 04103 Leipzig, Germany; 2 Uganda Wildlife Authority, PO Box 3530, Kampala, Uganda; 3 International Gorilla Conservation Program, P.O. Box 931, Kigali, Rwanda; 4 Present Address: Animal Ecology, Department of Ecology and Genetics, Evolutionary Biology Centre, Uppsala University, Norbyvägen 18D, 752 36 Uppsala, Sweden

**Keywords:** Dispersal, Philopatry, Solitary male, Dominance transitions, Alpha status

## Abstract

Dispersal is a key event in the life of an animal and it influences individual reproductive success. Male mountain gorillas exhibit both philopatry and dispersal, resulting in a mixed one-male and multimale social organization. However, little is known about the relationship between male dispersal or philopatry and reproductive careers in Bwindi mountain gorillas. Here we analyze data spanning from 1993 to 2017 on social groups in Bwindi Impenetrable National Park, Uganda to examine the proportion of males that disperse, age of dispersal, pathways to attaining alpha status, fate of dispersing males and philopatric males, and male tenure length as well as make comparisons of these variables to the Virunga mountain gorilla population. We report previously undocumented cases of dispersal by immature males and old males and we also observed the only known case of a fully mature male immigrating into a breeding group. We used genetic tracking of known individuals to estimate that a minimum of 25% of males that disperse to become solitary males eventually form new groups. No differences were found between the Bwindi and Virunga population in the age of male dispersal, the proportion of males that disperse, the age of alpha male acquisition, and dominance tenure length. The lack of differences may be due to small sample sizes or because the observed ecological variability does not lead to life history differences between the populations. Males in both populations follow variable strategies to attain alpha status leading to the variable one-male and multimale social organization, including dispersal to become solitary and eventually form a group, via group fissioning, usurping another alpha male, or inheriting the alpha position when a previous group leader dies.

## Introduction

Dispersal is a key event in the life of an animal because of the linkages to population dynamics, life history strategies, genetic and social structuring of a population, and variance in reproductive success (Dobson 2013; Greenwood 1980). Dispersal decisions and dispersal distance are influenced by many factors including reproductive opportunities, risk of inbreeding, competition for resources, and ecological conditions (Clutton-Brock 2016; Gilroy and Lockwood 2012). Furthermore, the future success of dispersing individuals may vary, depending on the age at dispersal as well as the opportunities to acquire mates outside of the natal group (Clutton-Brock 2016; Davidian et al. 2016).

In social living species, such as primates, dispersal patterns result in within- and between-species variation in social organization (Chapman and Rothman 2009; Kappeler and van Schaik 2002; Pusey and Packer 1987). Despite most primate species exhibiting universal male dispersal (Pusey and Packer 1987), some species have universal male philopatry (Furuichi et al. 2015) or both philopatry and dispersal (Chowdhury et al. 2015; Koenig and Borries 2012; Ostro et al. 2001; Port et al. 2012; Sterck 1999). In primates, components of maturation and male dispersal patterns can have implications for the reproductive careers and variance in reproductive success of individual males (Alberts 2012; Alberts and Altmann 1995; Port et al. 2012; van Noordwijk and van Schaik 2001, 2004). For example, in societies where male rank influences reproductive success, dispersal strategies may be determined by the viability of attaining the alpha position via tactics such as challenging a current dominant male, forming a new group by attracting females, or a group fission (van Noordwijk and van Schaik 2004).

Mountain gorillas are an interesting species in which to examine patterns of male dispersal and reproductive careers because they exhibit both philopatry and dispersal, which may lead to variation in male reproductive success (Harcourt 1978; Robbins 1995; Robbins et al. 2009a, b; Stoinski et al. 2009a). In contrast, male western gorillas appear to have universal male dispersal (Robbins et al. 2016). Because multimale groups form as a result of male philopatry and not takeovers by outsider males, multimale groups in western gorillas are extremely rare in comparison to both mountain gorilla populations that contain about 40% multimale groups (Robbins et al. 2016; Robbins, 1995; Robbins et al. 2009b). Variation in male dispersal patterns has implications for group structure and possible variation in male tenure length and reproductive success between the two species (Robbins and Robbins 2018).

**Fig. 1:**
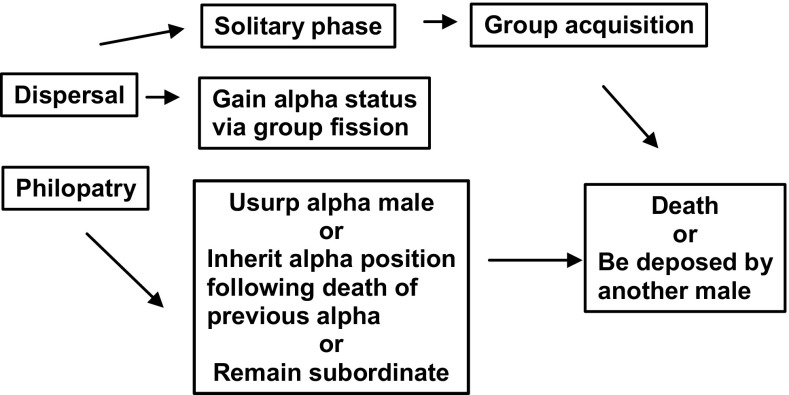
Trajectory of male reproductive career for mountain gorillas

For male mountain gorillas, the first decision of their reproductive career is whether to disperse or remain philopatric (Fig. 1). Dispersal includes males who emigrate to become solitary, as well as males who leave their group with other gorillas as part of a group fission (Stoinski et al. 2009a). Dispersal also includes involuntary transfers, in which an immature male joins a new group following the disintegration of his previous group, typically due to the death of the silverback. Solitary males may subsequently form one-male groups by attracting females from established groups. Philopatric males may become alpha by usurping the current alpha male, inheriting the alpha position following the death of a previously alpha male, or alternatively they can remain subordinate (Fig. 1). In the Virunga mountain gorilla population, philopatric males had higher lifetime reproductive success than dispersing males, suggesting that philopatry appears to be a more beneficial strategy, yet some males still disperse (Robbins and Robbins 2005; Watts 2000). The probability of male emigration does not appear to be influenced by the number of potential mates in the group, the number of potential competitors, or the age of dominant male (Stoinski et al. 2009a; Watts 2000). Instead, mating opportunities and the presence of mothers may lead to male philopatry (Robbins et al. 2016; Stoinski et al. 2009a, b).

The habitats and diets of the two remaining populations of mountain gorillas (Virunga Massif and Bwindi Impenetrable National Park, Uganda) are different, largely due to altitudinal variation (Robbins et al. 2006b; Wright et al. 2015). The mountain gorillas in the Virunga Massif consume almost no fruit because there is so little available and nearly all of their dietary items are available year-round. In contrast, Bwindi is lower altitude, so it contains more seasonally available (and less predictable in availability) fruit and the gorillas’ diet is roughly 10–15% fruit. The ‘risk aversion hypothesis’ predicts that an increase in the seasonality of food resources leads to slower life histories (Janson and van Schaik 1993). The risk aversion hypothesis was developed to explain the slow life histories of primates in general, as well as variation in life history parameters within and between species. It is based on the premise that mortality risk for juvenile primates is high due to risk of starvation and predation, resulting in slow growth rates to reduce the risk of starvation, such that as variation in food availability increases, the risk increases, so slower growth is expected (Janson and van Schaik 1993). Life history studies have shown that ecological variation can explain variation in female reproductive rates, age of first reproduction, and adult lifespan (Onyango et al. 2013; Pusey 2012), but the same principles could apply to male dispersal and reproductive careers (Alberts 2012). The ecological variation between Bwindi and the Virungas could explain why the interbirth interval is 5 years in Bwindi compared to 4 years in the Virungas (Robbins et al. 2009b; Wright et al. 2015). The inter-population ecological variation could also lead to variation in certain life history traits such as age of maturation, the proportion of males that disperse, the age of dispersal and alpha rank acquisition, and tenure lengths. Longer alpha male tenures could indicate a slower life history, or differences in the intensity of mating competition.

Relatively little is known about the life history patterns of male mountain gorillas in Bwindi (Robbins et al. 2009b), so the aims of this paper are to characterize the dispersal patterns and reproductive careers for Bwindi mountain gorillas and compare these with published information from the Virunga mountain gorillas. Specifically, we quantify the age and proportion of dispersing immature and mature males, the fate of solitary males, age of acquiring alpha male status and tenure length. We used the long-term demographic database from the habituated gorilla groups in Bwindi spanning 1993–2017. Furthermore, assessing the fate of solitary males, specifically when and if they form a group or die, is notoriously difficult because it is not feasible to continue to monitor them over a long time period. Therefore we use genetic data obtained from the 2006 and 2011 mountain gorilla censuses to track whether the emigrant males were still solitary after 5 years or had attained a group (Guschanski et al. 2009; Roy et al. 2014).

## Methods

### Study population and data collection

**Fig. 2:**
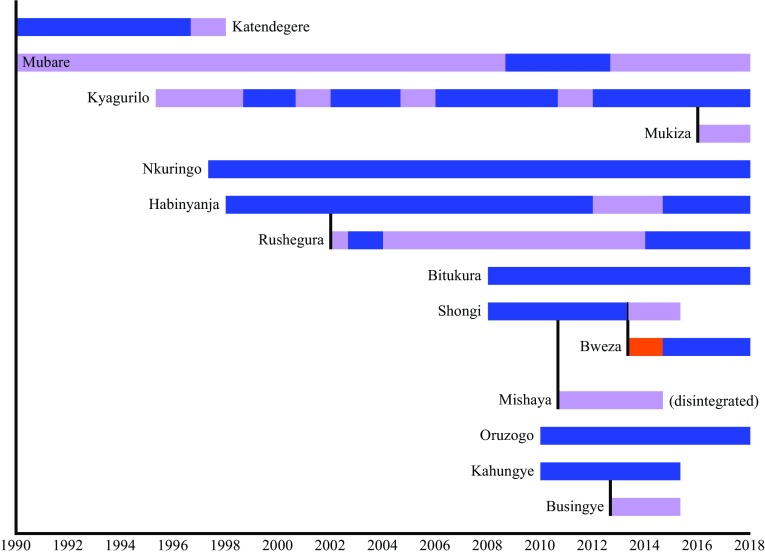
Timelines of groups included in the study. The year of the study is denoted on the *x*-axis. Each bar on the year axis represents a different study group and when they were observed. Blue coloring indicates when groups were multimale and purple indicates when they were one-male. Orange denotes when a group was an all-male group. Solid black vertical lines indicate when a group fissioned into two groups

This study was conducted in Bwindi Impenetrable National Park, located in the southwest corner of Uganda (0°53′–1°08′N; 29°35′–29°50′E). The “main” dataset includes information that was collected from 1993 to 2017 from seven gorilla groups that have been habituated for tourism and research, and two solitary males who were monitored for a few months after leaving their groups (Fig. 2). Four groups (Ka, Mu, Ru, and Haa) ranged around Buhoma in the western section of the park, two groups (Ky and Bit) ranged near Ruhija in the eastern section of the park, and one group (Nk) ranged in the southwest (Robbins et al. 2009b; Seiler et al. 2018). These groups were monitored daily, allowing for accurate recording of all births, deaths, and dispersal events. In addition to the main dataset, an “expanded” dataset includes six other habituated groups that were monitored from 2008 to 2017, but the exact group compositions were not known and demographic events were not systematically recorded (Fig. 2). Those groups are used only in analysis of dominance tenures.

For this study, we used the same age categories as those used for Virunga mountain gorillas (Robbins et al. 2006a). Infants are defined as those gorillas < 3 years old, juveniles are 3–5 years of age, and subadults are 6–7 years old. Females are considered adult at age 8 years. Males between 8 and 11 years are classified as blackbacks, and those 12 + years old are called adult males or silverbacks. Immatures are defined as the sum of infants, juveniles, and subadults (Kalpers et al. 2003). Because many gorillas were born before their groups were habituated, the exact birth dates are not known for all individuals. For any analyses using the age of males, we restrict the dataset to individuals whose birthdate was estimated to the nearest 6 months (Robbins et al. 2009b).

Emigration was defined as the age when the male “was no longer observed in their group and had ceased making repeated visits to the group lasting more than 1 day” (Stoinski et al. 2009a). An involuntary transfer occurred when a male joined a new group following the disintegration of his previous group, typically due to the death of the silverback. Dispersal included males who emigrated to become solitary and those who left in a group fission. Philopatry included males who died or became dominant in the group after reaching age 12. Philopatry does not include males who were still subordinate at the end of observations because it was not known whether they would subsequently disperse, become dominant without dispersal, or remain subordinate until their death. All statistical results for young silverbacks are based on the assumption that unexplained disappearances were due to emigration. Results were similar when we assumed that the unexplained disappearances were due to death.

In addition to direct observations of habituated groups, we also used genotype data from population-wide censuses to track individuals that were found as solitary males or in groups in the 2006 census and subsequently found in the 2011 census as solitary males or in a different social unit (Guschanski et al. 2009; Nsubuga et al. 2008; Roy et al. 2014). The number of gorillas in Bwindi Impenetrable National Park was estimated in 2006 and 2011 by means of ‘sweep’ census efforts to survey the entire park for detection of ‘nests’ constructed in the evening by individual gorillas (Guschanski et al. 2009; Roy et al. 2014). Dung left in the nests was collected for genetic analysis, and gorilla counts were based on a combination of nest count data and genetic data. DNA extracted from the fecal samples was used to produce individually specific genetic profiles by means of microsatellite genotyping, as previously detailed. We compared all genotypes from the 2006 and 2011 censuses to detect movement of males from one social group to another, or from solitary to social group or vice versa.

### Statistical analyses

We used a generalized linear mixed model (GLMM; Jiang 2007) to compare the ratio of dispersal:philopatry among young silverbacks at Bwindi versus the Virungas. The model was run with a binomial error structure and logit link. The analysis used one data point for each male who dispersed or remained philopatric during the study. The response variable equaled “1” if the male dispersed and “0” if he was philopatric. The predictor variable was the gorilla population, and the model included a random effect variable for the group that the gorilla left.

To determine if the proportion of males that emigrated to become solitary differed between populations, we used a similar GLMM to compare the ratio of males who emigrated versus those who became dominant without emigration (e.g., males that formed a group via fissioning were not considered emigrants). The response variable equaled “1” if the male emigrated and “0” if he became dominant without emigrating to become solitary. Males could become dominant by inheriting or usurping the dominant role, or through group fission. We excluded males that did not remain dominant long enough to sire any offspring.

To compare the age of emigration between Bwindi and the Virungas, we ran a GLMM with one data point for each emigrant, with the response variable being the age of emigration. The predictor variable was the gorilla population, and the model included a random effect variable for the group that the gorilla left.

To compare the ages at which subordinate males became dominant, we ran a GLMM with one data point for each young silverback who inherited or usurped the dominant role in his group, as well as males who became dominant through a group fission. The analysis excluded solitary males who became dominant by acquiring females because such males are not monitored routinely and we do not have data on when they became dominant. The response variable was the age of becoming dominant; the predictor variable was the gorilla population; and the model included a random effect variable for the group where the gorilla had been subordinate.

To compare the probability of remaining subordinate (versus age), we used a mixed effect Cox model (Pankratz et al. 2005). The analysis used a separate data point for each male who was observed to reach age 12. The predictor variable was the gorilla population, and the model included a random effect variable for the group where the gorilla had been subordinate. Uncensored data points equaled the normalized age when males emigrated, became dominant, or died. Data points were censored if males were still subordinate when the study ended. The Bwindi data were compared with published results from Karisoke study area of the Virungas (Stoinski et al. 2009a) that included males living in a mixed-sex group at the time they became silverbacks and whose birthdates were estimated to the nearest month (12 years of age; *N* = 31).

Using the genetic tracking data, we investigated if success rates of solitary males forming groups increased over time, which would suggest that they need to continue maturing after dispersal, as Karisoke males continue to grow until about 16 years of age (Galban et al. 2017). Conversely, if the success rate declined over time, it might suggest that high-quality males succeed more quickly than lower quality males. To do this, we performed a Fisher’s exact test to compare the frequency of males that were solitary in 2006 and in a mixed sex group in 2011 (presumed dominant male) versus males who were in a social group in 2006 but found in another social unit in 2011. A higher proportion of males who were solitary in 2006 and had groups in 2011 than males who were in groups in 2006, became solitary, and were in a different group in 2011 would indicate that males need time to mature after emigrating (the former were solitary for a longer period of time than the latter), whereas if the opposite held it would indicate that high-quality males acquire groups more quickly than low-quality males.

We used rate-based *χ*^2^ calculations to compare the rates of terminations of dominant males in Bwindi versus the Virungas (Altmann and Altmann 1977). The termination rate equaled the number of dominant male terminations in each population (deaths plus usurpations), divided by the collective number of years that dominant males were observed in each population. Male tenure length can be estimated as the inverse of the termination rate (Dunbar 1984; Makarieva and Gorshkov 2004).

We also used rate-based *χ*^2^ calculations to compare the mortality rates for dominant males in each population. The mortality rate equaled the number of dominant males that died in each population, divided by the number of group-years that were observed in each population (Janson and van Schaik 2000). The expected number of deaths was proportional to the number of male-years that each population was observed. Male-years equal the number of elapsed days from the beginning to the end of observations for each male, divided by 365.25. These analyses included silverbacks of unknown age but excluded males that did not remain dominant long enough to sire any offspring (e.g., solitary males that acquired females for a few days or months before losing them again).

All statistical analyses were performed in R, except for the rate-based *χ*^2^ calculations which were performed in Excel. Results were considered statistically significant if the *p* value was less than 0.05.

## Results

### Immature males

The main dataset contains five inter-group transfers by three immature males whose ages ranged from 6 to 12 years old. However, two of the males returned to their natal group (7 and 9 months later), so these were not permanent dispersal events. The remaining three dispersing immature males represent 4.4% of the 68 males who were observed in the study groups between the ages of 6–12. In addition, one male immigrated from an outside group at an estimated age six. There were no known cases of involuntary transfers by immature males in the main dataset, but only one of the study groups disintegrated at the very end of the study. One group disintegrated in the expanded dataset, and at least one immature male (age 9) made an involuntary transfer into a non-breeding group (along with three adult females and an infant).

### Subordinate males

Among the 31 subordinate males who reached age 12 during the study, eight males (26%) were still subordinates when observations ended. Among the other 23 males, 19 emigrated to become solitary (83%), three inherited the dominant role (13%), and one become dominant during a group fission (4%).

Considering emigrant males as those that became solitary and those that acquire groups via fissions as having dispersed, the 20:3 ratio of dispersal:philopatry at Bwindi was not significantly different from 15:7 ratio in the Virungas (LRT = 1.48, *df* = 1, *p* = 0.22). To examine if the proportion of males that became solitary differed between populations, we used a similar GLMM to compare the ratio of males who emigrated versus those who became dominant without emigration (males that become dominant via fissions were not considered as emigrants). The 19:4 ratio of emigration versus becoming dominant at Bwindi was not significantly higher than the 8:8 in the Virungas (LRT = 2.51, *df* = 1, *p* = 0.11).

**Fig. 3:**
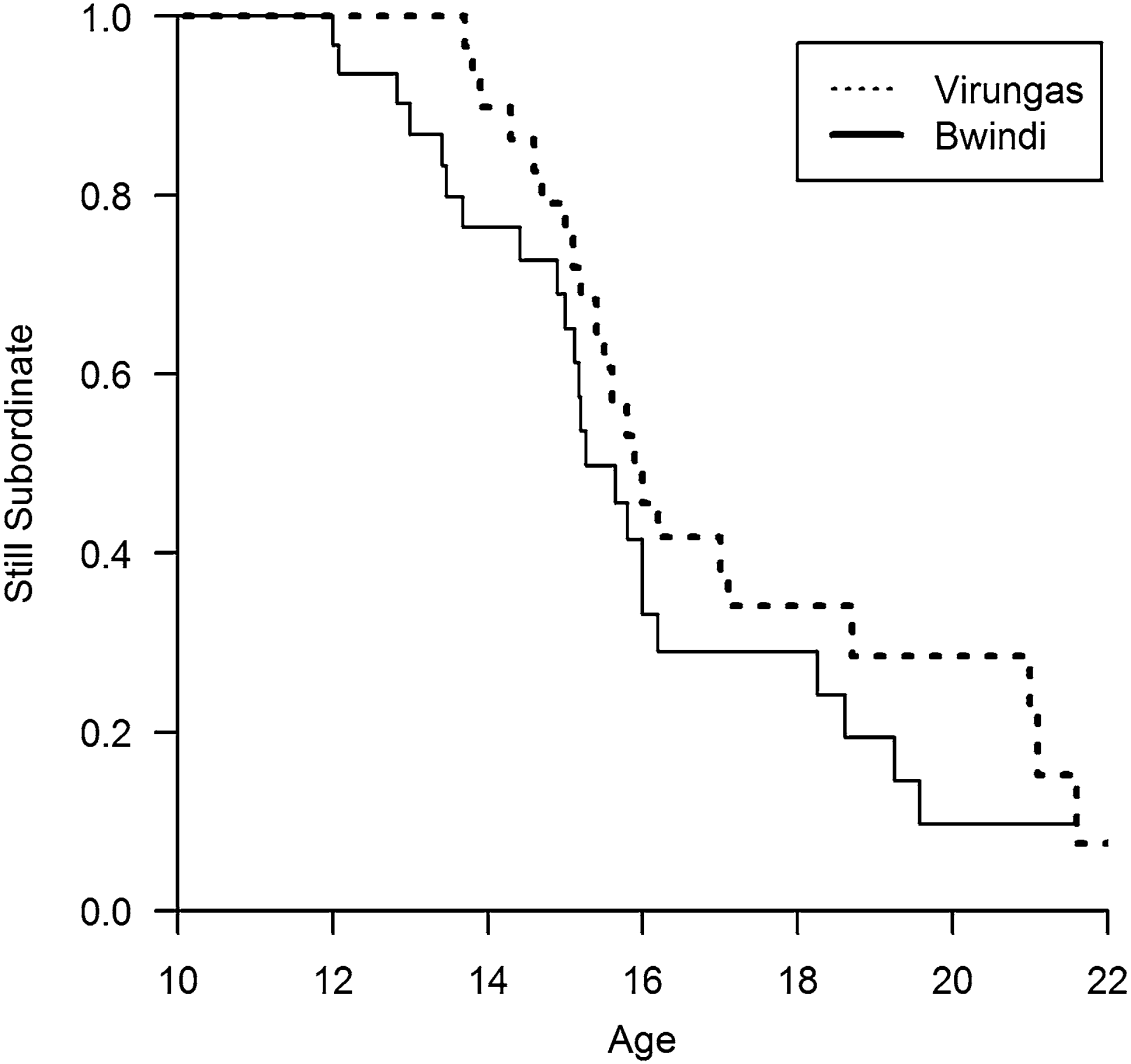
Probability (versus age) for males to remain subordinate in the Virungas (dotted line) versus Bwindi (solid line)

The average age of emigration was 14.3 ± 1.0 at Bwindi (n = 7), which is not significantly different from 15.7 ± 2.3 years for eight males in the Virungas (LRT = 1.68, *df* = 1, *p* = 0.19). The average age of becoming dominant was 15.0 ± 1.7 years at Bwindi (n = 3), which is not significantly different from 16.7 ± 2.2 years for eight males in the Virungas (LRT = 1.78, *df* = 1, *p* = 0.18). The probability of remaining subordinate (versus age) was significantly lower at Bwindi than in the Virungas (coefficient = − 0.95, *z* = *−* 2.09, *p* = 0.036), suggesting that Bwindi males were more likely to either emigrate or become dominant at a younger age than in the Virungas (Fig. 3). Less than 10% of the males remain subordinate past age 18 at either site, which suggests that philopatry is not a common strategy unless the male becomes dominant.

### Fate of solitary males

The census data from 2006 and 2011 provides information on the fate of solitary males in two ways. First, we considered a cohort of 13 males who were identified in a breeding group in 2006 and were then found in a different social unit in 2011 (Table 1). Five of those 13 males were leading a new group in 2011, which suggests a 38% initial success rate of group formation for dispersing silverbacks. The other eight males were solitary in 2011. Second, we identified ten solitary males in the 2006 census, of which six were still found in 2011. Only one of those six males had formed a new group (17%), and the other five were still solitary. The success rate of group formation for those six emigrants is not significantly different from the 13 males who were previously identified in a group (Fisher’s exact test, *p* = 0.34), providing no evidence for the predictions that solitary males either acquire groups quickly or males require time to reach quality to attain groups. The six new groups contained an average of 4.5 ± 2.0 gorillas. All of the groups contained at least one female, and only one group contained more than one male.

**Table 1:** Genetic tracking of solitary and dispersing males from 2006 to 2011

2006	2011
In breeding group (13)	Group leader = 5
	Solitary = 8
Solitary (10)	Group leader = 1
	Solitary = 5
	Not found = 4

Thirteen males that were in breeding groups in 2006 were tracked genetically in 2011 to be either group leaders or solitary. Ten males found as solitary in 2006 were found in 2011 as a group leader, solitary or not found

### Dominance transitions

After including groups when the entire composition was not fully known (Fig. 2), the expanded database includes portions of 25 dominance tenures. Nine of the dominance tenures had already begun when the groups were initially observed. Using the remaining 16 tenures to determine how they began, nine began when the previous alpha male died (56%), five started when the group fissioned (31%), and the other two began when a subordinate usurped the dominant position (13%). One case of attaining alpha status (after the alpha male died) was by a male who immigrated into a breeding group as a fully mature silverback, which is the first reported case of such an immigration. To determine how tenures ended, 12 of the dominance tenures were ongoing at the conclusion of this study, so we considered the other 13 tenures. Eleven tenures ended when the dominant male died (85%) and the other two dominant males were usurped (15%). Both of the usurped males became solitary after losing their alpha status; they subsequently died within 5 months of being usurped. In one case, the silverback had a badly injured leg that likely prevented him from keeping up with the group. Additionally, two visibly old subordinate males, who may have been deposed prior to the habituation of the group, repeatedly emigrated out of and immigrated into their group for more than 2 years.

The 11 deaths of dominant males occurred during 157.6 years of observed tenures, which represents a mortality rate of 0.070 deaths per year for dominant males. A mortality rate of 0.048 deaths per year has previously reported for dominant males in the Virungas (Robbins et al. 2013), which is not significantly different from the results from this study (rate-based Chi-square = 0.87, *df* = 1, *p* = 0.35). These results suggest that adult lifespans are not significantly longer for dominant males at Bwindi versus the Virungas.

Dominance tenures ended at a rate of 0.082 terminations per year, which is not significantly different than the corresponding rate of 0.064 for the Virungas (rate-based Chi-square = 0.54, *df* = 1, *p* = 0.46). The reciprocal of the termination rate equals 12.1 years at Bwindi (versus 15.7 years in the Virungas), which represents an estimate of the average dominance tenure length.

## Discussion

Despite ecological variation between the two mountain gorilla populations and a longer interbirth interval in Bwindi compared to the Virunga Volcanoes (Robbins et al. 2009b), we found no difference between the two populations in the age of male dispersal, age of attaining alpha male rank, or male tenure length. A larger proportion of males emigrated to become solitary in Bwindi (83%) than in the Virungas (about 50%), but a larger sample size may be needed to detect a statistically significant difference. The lack of differences in age of dispersal and attaining alpha status is somewhat unexpected because the longer interbirth interval in Bwindi males would suggest a later weaning age and that they might attain full body size at a later age, leading to a delay in these life history markers. However, Bwindi males may not differ in their growth trajectories, they may compensate somehow in their growth trajectories during maturation, or Bwindi males may be smaller than Virunga males at maturity. An ongoing study examining body size and growth trajectories in the two populations will help resolve this issue. Similarly, the mortality rates of dominant males at Bwindi was not significantly higher than the Virungas, suggesting that adult lifespans are not significantly longer for dominant males at Bwindi versus the Virungas, so they do not provide evidence of a slower life history at Bwindi.

Alberts and Altmann (1995) suggest that a two-level process influences variance in life history traits and lifetime fitness of male baboons, specifically deterministic processes including maternal effects and demographic factors such as female availability and other competitive males, with stochasticity playing a larger role in the latter. In other words, environmental and/or maternal factors may affect the variation in age of maturation and survivorship, but the age of attaining dominance and reproductive success may be due to the opportunities available during a certain time window depending on the strength and number of competitors as well as the availability of females (Alberts and Altmann 1995; Kramer et al. 2017; van Noordwijk and van Schaik 2004). A similar pattern may be occurring in male mountain gorillas, with several pathways to attain alpha status, notably dispersal or philopatry, and female choice may play a strong role (see also Steenbeek et al. 2000). Females’ choice of males via female dispersal is likely to be influenced by male size and strength (Breuer et al. 2012). However, females will be constrained by which males are available in the nearby vicinity since they only transfer during intergroup encounters with neighboring groups and during the limited time window when they are not pregnant nor have unweaned offspring vulnerable to infanticide (Robbins et al. 2009a, b).

Alpha rank acquisition by philopatric males may occur via usurpation or the death of the previous alpha male and some dispersing males become alpha via a group fission. Of the 16 males who acquired alpha status in Bwindi via those routes, 56% inherited it, 31% acquired it through group fission, and 13% usurped the previous alpha male. In comparison, of the eight males reported to become alpha at Karisoke (Stoinski et al. 2009a), 25% inherited it, 37.5% became alpha via a group fission, and 37.5% via usurpation. Given the small sample sizes, there is no apparent trend for one strategy being favored over another in either population, but it is apparent that males use all three tactics in both populations. Usurpation (challenges) was predicted to be the primary route to dominance acquisition for species with high paternity concentration (alpha male monopolizing most sirings) (van Noordwijk and van Schaik 2004), but in contrast, we found that usurpation was the least common tactic and that most dominance tenures ended when the male died. However, our results do support their prediction that males obtaining the alpha position via usurpation should be in their prime (van Noordwijk and van Schaik 2004), whereas males that become alpha following the death of the previous alpha male (succession) may have lower fighting ability (van Noordwijk and van Schaik 2004). If the alpha male in a multimale group dies, especially if due to stochastic events, a subordinate male may acquire alpha status sooner than he would if he had attempted to usurp the previous alpha male, which occurred twice in this study. One alpha male (RC in Kyagurilo Group) was killed by lightning and the alpha male of another group died after falling more than 20 m from a tree (ND in Bitukura Group). In the first case (Kyagurilo Group), female choice further influenced the reproductive success of the new alpha male (RR, estimated to be in his mid-20’s) as the group fissioned 1 year later, with four of seven females remaining with a young silverback (16 years old). Additionally, two females transferred out of RR’s group in 2016, leaving him with only one adult female and many juveniles that he did not sire.

Attaining alpha status leads to large reproductive payoffs in mountain gorillas because males sire all offspring in one-male groups and roughly 85% in multimale groups (Bradley et al. 2005; Nsubuga et al. 2008; Vigilant et al. 2015). Average tenure length is 12–15 years in both populations, with some males remaining dominant for 20 years or more (Robbins et al. 2013; Vigilant et al. 2015; this study). Therefore, male mountain gorillas have high monopolization potential for time periods spanning two, three, or more interbirth intervals.

We still lack sufficient data to determine how much demographic conditions and stochasticity influences the variance in the frequency of philopatry versus dispersal. Previous studies have struggled to explain which subordinate silverbacks will emigrate versus remain philopatric (Robbins 1995; Stoinski et al. 2009a; Watts 2000). Our current analyses suggest an answer: nearly all males emigrate unless they become dominant. Less than 10% of the males remain subordinate past age 18 at either site (Fig. 3), which suggests that philopatry is not a common strategy unless the male becomes dominant. From that perspective, mountain gorillas differ from species such as chimpanzees where philopatric males can remain subordinate for all/most of their adult lives.

The prospects for male mountain gorillas to become dominant probably depend on factors such as the relative strength of the males in the group, as well as random events that lead to the death of the dominant male. That may be one explanation for why quantitative factors such as group composition have been weak predictors of whether a male will emigrate (Stoinski et al. 2009a). Furthermore, subordinate males with more mating opportunities are more likely to remain philopatric (Stoinski et al. 2009b), suggesting that females can assess male quality and they preferentially mate with males who have a higher chance of becoming/remaining dominant. It is also not surprising that emigrants have lower reproductive success than philopatric males because if males emigrate unless they become dominant, then philopatric males will almost always have reproductive success (whereas emigrants have no such certainty of acquiring females). If emigrants are the males who could not become dominant in their natal group, then they may typically have lower quality than the (philopatric) males who could become dominant. Thus the emigrants may merely be making the most of a bad job, and their lower success does not necessarily indicate that emigration is a poor reproductive strategy (Robbins and Robbins 2018).

The genetic tracking of dispersing males in this study provides some of the first information on the ability of these males to acquire mates and form new groups (see Hagemann et al. 2018 for western gorillas). Assuming that males did not briefly acquire females and then return to a solitary state if they all dispersed again, our results show that males may be solitary for as much as 5 years, suggesting either that they need a significant amount of time to continue developing before they are able to acquire groups or that there are few opportunities for these males. However, two of those silverbacks emigrated from a habituated group at ages 13 and 15, and remaining solitary for less than 1–2 years, which illustrates that emigrants can form new groups relatively quickly and at relatively young ages, and further stresses the variability in male success. Our results about solitary males are comparable to those from Karisoke in which 27% of dispersing males successfully formed groups (three out of 11 males whose fate was known 1–112 months after dispersing; Stoinski et al. 2009a). The results from both populations lend support to the results from modeling the Virunga population that dispersing males have lower reproductive success than philopatric males (Robbins and Robbins 2005).

Lastly, we also observed several novel events involving male dispersal. Specifically, immature males transferred into other heterosexual groups for some months and eventually return to their natal group. It is unclear why these males dispersed, but anecdotal evidence suggests that during intergroup encounters they may have been interacting with members of the other group and simply did not follow their natal group when the two groups separated at the end of the encounter. Nonetheless, these dispersal events by immature males may be one explanation for the occurrence of unrelated males co-residing in a group (Bradley et al. 2005). Two usurped males ‘emigrated’ from their group and died after a few months of being solitary. We also observed older, presumably deposed silverbacks repeatedly becoming solitary and rejoining their group, possibly because they could not always keep up with the group. Such events involving older silverbacks have not been documented in the Virungas and these cases show that not all solitary males in a population are in good enough physical condition to form new groups. We also report the first known case of a fully mature silverback immigrating into an established heterosexual group. These novel observations stress the value of long-term field studies for obtaining a comprehensive understanding of the flexibility in male life history strategies and primate grouping patterns (Clutton-Brock and Sheldon 2010; Janson 2000; van Noordwijk and van Schaik 2004).
